# The Influence of Physical Load on Dynamic Postural Control—A Systematic Replication Study

**DOI:** 10.3390/jfmk5040100

**Published:** 2020-12-21

**Authors:** Jessica Heil, Sarah Schulte, Dirk Büsch

**Affiliations:** Institute of Sport Science, Carl von Ossietzky University Oldenburg, Ammerländer Heerstraße 114-118, 26129 Oldenburg, Germany; sarah.schulte@uol.de (S.S.); dirk.buesch@uol.de (D.B.)

**Keywords:** fatigue, injury risk, side-differences, Y-Balance Test

## Abstract

Dynamic postural control is challenged during many actions in sport such as when landing or cutting. A decrease of dynamic postural control is one possible risk factor for non-contact injuries. Moreover, these injuries mainly occur under loading conditions. Hence, to assess an athlete’s injury risk properly, it is essential to know how dynamic postural control is influenced by physical load. Therefore, the study’s objective was to examine the influence of maximal anaerobic load on dynamic postural control. Sixty-four sport students (32 males and 32 females, age: 24.11 ± 2.42, height: 175.53 ± 8.17 cm, weight: 67.16 ± 10.08 kg) were tested with the Y-Balance Test before and after a Wingate Anaerobic Test on a bicycle ergometer. In both legs, reach distances (anterior) and composite scores were statistically significantly reduced immediately after the loading protocol. The values almost returned to pre-load levels in about 20 min post-load. Overall, findings indicate an acute negative effect of load on dynamic postural control and a higher potential injury risk during a period of about 20 min post-load. To assess an athlete’s sports-specific injury risk, we recommend testing dynamic postural control under loaded conditions.

## 1. Introduction

Balance or postural control can be prescribed as a human’s ability to resist perturbations and maintain stability [1,2]. It is distinguished between static and dynamic postural control and balance. Static balance is defined as “the ability to maintain the center of gravity within a base of support in a quiet upright position during standing or sitting” (p. 322). Whereas “dynamic balance involves maintaining an upright posture while (a) the center of gravity and base of support are moving and (b) the center of gravity is moving outside the base of support (for example, in walking)” [2] (p. 322). This also involves the “maintenance of balance while transitioning from dynamic to a static state” [3] (p. 103). Therefore, dynamic postural control is important in many sports where dynamic actions such as landings or cuttings are present and require reactive and compensatory movements [4,5]. In these situations, non-contact injuries such as anterior cruciate ligament (ACL) ruptures or ankle ligament injuries mainly occur [6,7,8]. A decrease of dynamic postural control is one factor that is associated with a higher injury risk for lower-limb non-contact injuries [3,5]. Therefore, the testing of dynamic postural control could give information about an athlete’s potential injury risk [9,10,11,12]. Most of the time, dynamic balance, similar to other risk factors, is tested under resting conditions. However, non-contact injuries occur under loading conditions during matches or competitions as well as in sports training [13]. Hence, risk factors, such as dynamic postural control, should not only be regarded under resting conditions but also be investigated under the influence of load to assess an athlete’s injury risk and to reveal possible deficits that are not apparent during rest [3,14]. 

The majority of studies regarding the influence of load on postural control tested subjects under static conditions [4,15]. These studies indicate a negative effect of load on static postural control. However, several studies also examined the influence of physical load on postural control under dynamic conditions. Several studies indicate that physical load seems to have a negative effect on dynamic postural control [3,15,16,17,18], leading to an increased injury risk [5,16,19,20]. For example, Johnston et al. [3] found a significant decrease of dynamic postural control after a cycling protocol. Similarly, Gribble et al. [15] revealed a decrease of dynamic postural control after local fatiguing protocols, whereas Whyte et al. [5] showed a negative effect of a high-intensity training (HIT) protocol on dynamic postural control. Moreover, Sarshin et al. [17] found a negative effect of a functional loading protocol on dynamic postural control in badminton players. These studies were all using the Star Excursion Balance Test (SEBT) or the Y-Balance Test (YBT) to assess dynamic balance. Additionally, Wright et al. [20] and Güler, Aras, Akça, Bianco, Lavanco, Paoli, and Şahin [21] found a negative effect of running and cycling on dynamic postural control measured with a Biodex Balance System. This load-induced decrease in dynamic postural control is supposed to be due to a loss of sensorimotor control and neuromuscular impairments [4,5,22,23] resulting from changes of the afferent information, altered proprioception, and changes of joint awareness and stability [18,23,24,25,26,27,28,29]. Moreover, physical activity might also increase stress levels and affect executive functions of an athlete, both influencing postural control [30,31]. 

The pattern of evidence, however, is inconsistent and partly conflicting, and there are also studies not proving an effect of physical load on dynamic postural control. For example, Wright et al. [20] found a negative effect of a loading protocol on a treadmill, but no effect after a loading protocol on a cycling ergometer. Likewise, Zech et al. [4] found no effect of physical load on dynamic postural control in healthy handball athletes. This inconsistency in the results might be due to the different methods used, and different tests might not challenge postural control in the same way [3] or challenge different aspects of dynamic postural control, i.e., reactive vs. anticipatory postural control [2]. Moreover, different systems (sensorimotor system, vestibular system, visual system, etc.) might not be stressed equally by the loading protocols [20,29], and the protocols might induce different types of fatigue (whole body vs. local) [20]. Nevertheless, loading protocols should reflect sport-specific demands and situations in order to be able to make reliable and consistent statements [32,33]. 

Moreover, the results are influenced by other factors such as expertise [16], gender [5,15], or injury history [4,22,27]. To exemplify, Baghbani et al. [16] found an effect of a seven-station exertion protocol on dynamic postural control in female non-athletes but not in female athletes. Greater impairments of dynamic postural control after running on a treadmill was found in subjects with a history of ankle sprains [22] and chronic ankle instability [27] compared with healthy controls. In another study, Gribble et al. [15] showed that females had better dynamic postural control than males and were less affected by local fatiguing protocols. Whyte et al. [5] found the same after a HIT protocol. As a consequence, these potential factors need to be controlled in research. 

Furthermore, both legs are involved in sporting movements, injuries can occur in both legs [34] and corresponding side-differences are an additional factor enhancing the likelihood for non-contact injuries [8,33]. However, most of the studies assessed dynamic postural control of only one leg [3,4,5,16,20,22], usually of the ‘dominant leg’, mostly defined as the athlete’s kicking leg [3,5] or the ‘take-off leg’ [4]. In studies with formerly injured athletes, mostly the injured leg was regarded [22]. Only a few studies considered side-differences. Gribble and Hertel [35] found no differences between the left and right leg and combined the results in further analyses. Moreover, Fusco, Giancotti, Fuchs, Wagner, da Silva, and Cortis [36] assessed the influence of leg dominance and found no difference between the dominant (kicking leg) and non-dominant leg (standing leg). However, they were testing dynamic postural control under resting conditions. Concerning the influence of load, Gribble et al. [27] compared the injured with the non-injured leg in subjects with chronic ankle stability after local fatiguing protocols and found greater decrease in the injured leg compared with the non-injured leg as well as compared with healthy controls. In another study, Gribble et al. [15] found no differences between the legs in healthy subjects after local fatiguing protocols. So far, however, the question of whether and how global fatiguing protocols affect side-differences in dynamic postural control remains open. 

In the context of injury prevention, it is also relevant (e.g., to derive practical implications for clinicians and coaches) to look at the time dynamic postural control needs to recover after a load [3]. Specifically, knowing about how long dynamic postural control is impaired after physical load to consider in both the assessment of injury risk and the conceptualization of training sessions and training programs. So far, only a few studies regarded the recovery time of dynamic postural control after physical load. In the study of Johnston et al. [3], dynamic postural control almost recovered in about 20 min post-load, whereas Wright et al. [20] display recovery times of only 9–12 min, and Güler et al. [21] found recovery times of about 10 min. These differences might be also caused by different tasks and protocols that were used [3], and to obtain more information on this further studies are needed. 

Altogether, no clear assertions can be made on the influence of load on dynamic postural control. Hence, several studies indicate that physical load might have a negative effect on dynamic postural control. Nevertheless, there are conflicting results and still many open aspects, and the effects need to be verified and confirmed in further studies using a systematic approach [5]. Therefore, the aim of the current study was to investigate the effect of physical load on dynamic postural control, to regard the effect for both legs and to examine the recovery time of dynamic postural control. 

Regarding the mentioned studies, there were methodological differences, and some of the used designs seem better at reflecting the physical demands of sport-specific actions. The study of Johnston et al. [3] showed good methodological quality to assess the influence of physical load on dynamic postural control. The used methods YBT [37,38,39] and Wingate Anaerobic Test [40], seem appropriate to reflect sporting demands. The YBT derived from the SEBT, which was often used in former studies, and became a reliable tool to measure dynamic postural control [37,38,39]. It depicts the demands of real sporting situations and gives information about the injury risk of an athlete [17,26,38] with the obtained composite score [6] or the side-difference in anterior direction [11]. The modified version of the Wingate Anaerobic Test [40] induces maximal anaerobic load during a short period of time and mimics short bouts of high intensity, as they frequently occur in sport, e.g., games such as soccer or handball [5]. For this reason, here the study design of Johnston et al. [3] was systematically replicated with a group of healthy sport students to control the stability of the effect. Moreover, the original study design was supplemented by investigations of the standing leg to compare the changes between the legs, and to check for potential side-differences. 

Based on the study of Johnston et al. [3], the following hypotheses were made. We hypothesized that dynamic postural control would decrease between the pre-load and the immediate post-load measurements (H1) and that dynamic postural control will successively increase after the load (H2). Moreover, we hypothesized that dynamic postural control would be at baseline levels 20 min post-load (H3). Additionally, the original design of Johnston et al. [3] was supplemented by investigations of the standing leg. We hypothesized that the aforementioned changes would occur in both legs (H4) and that (H5) potential side-differences will change due to the loading protocol. 

## 2. Materials and Methods 

The studies were conducted in accordance with the Declaration of Helsinki, and the local Ethics committee of the Carl von Ossietzky University Oldenburg, Germany approved the protocol (EK/2020/035-02, 24 June 2020).

### 2.1. Participants

The study was divided into two examination groups to control the sequence of testing. In Group 1, the subjects started the YBT standing on their kicking leg first, and in Group 2, the subjects started the measurements standing on their standing leg. 

For the one- and two-way repeated measures analyses of variance, a sample size of *n* = 61 respective *n* = 62 (F-Test: f = 0.25; α = 0.05, 1-β = 0.90) was determined a priori using G*power software (Vers. 3.1.9.7) [41] and the SPSS syntax by Wuensch [42] to determine the confidence interval of the effect size. In total, n = 64 sport students (32 males and 32 females, age: 24.11 ± 2.42, height: 175.53 ± 8.17 cm, weight: 67.16 ± 10.08 kg) participated in this study. The subject characteristics are provided in Table 1. All subjects had to be free of lower limb musculoskeletal injury in the previous six months. Moreover, participants with chronic ankle instability, vestibular or visual impairment, cardiovascular disease, previous reports of chest pain, neurological disease, and balance disorder, or taking medication for a balance disorder were not allowed to participate. Furthermore, all subjects had to complete the PAR-Q [43] before testing and were excluded when they answered ‘yes’ to any question.

### 2.2. Procedures 

All subjects participated in a 90-min session in a laboratory setting. Before testing, subjects were informed about the procedures and provided written informed consent to the following experiment. Moreover, they filled out a questionnaire including personal data, sporting background, injury history, and the PAR-Q [43] to check, among others, eligibility for inclusion in the loading protocol. Afterward, anthropometric measurements (weight, height) were recorded using the Inbody270 (InBody Co., Seoul, Korea) and a stadiometer (Seca GmbH & CO. KG, Hamburg, Germany). Leg length was measured with a measuring tape while the subject was standing in front of a wall. It was defined as the distance between the subject’s anterior–superior iliac spine and the most distal part of the medial malleolus [35,38]. 

**Figure 1:**
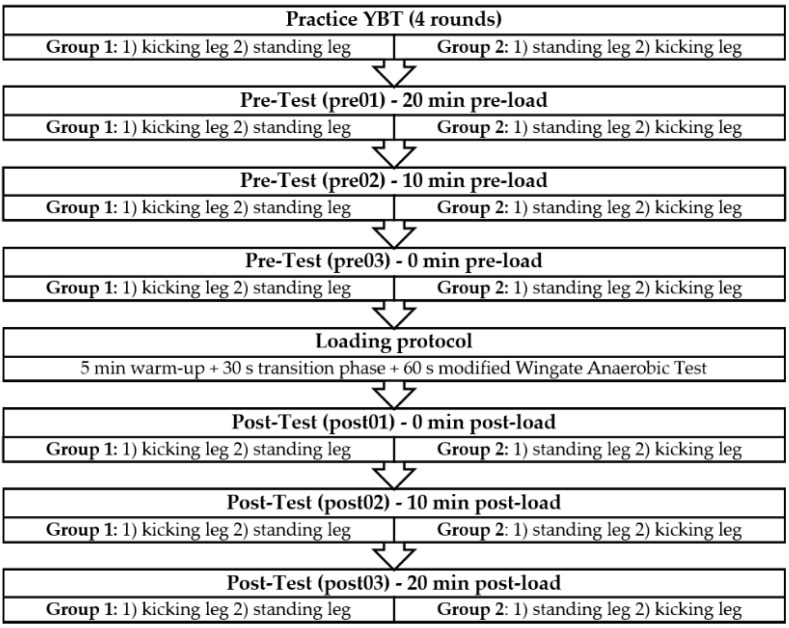
Study design.

Then, the design of Johnston et al. [3] was systematically replicated: First, subjects performed four practice rounds of the YBT to minimize possible learning effects [26]. One round consisted of a trial in all three directions of the YBT (anterior [ANT], posteromedial [PM], posterolateral [PL]) while standing on one leg, directly followed by a trial in all three directions standing on the other leg. One round had a duration of about 60 s. Group 1 started the round standing on their kicking leg, whereas Group 2 started on their standing leg. Next, three rounds of the YBT were recorded at baseline (20 min pre-load [pre01], 10 min pre-load [pre02], and immediately pre-load [pre03]), allowing reliability testing by computing the coefficient of variation (CV) [44] and intraclass correlation coefficients (ICC) [45]. Afterward, subjects completed a standardized 5-min warm-up with low-resistance cycling (male: 90 RPM, female: 60 RPM) on the ergometer and completed 3 × 5 s sprints during these 5 min. The warm-up directly merged into the 60 s Wingate Anaerobic Test. Throughout the protocol, the participants were verbally encouraged by the examiner to cycle as fast as possible. After completing the loading protocol, both groups directly went back on the YBT for the post-test measurements. All subjects performed three rounds of the YBT again, immediately post-load (post01), 10 min post-load (post02), and 20 min post-load (post03). A schematic study design is depicted in Figure 1.

### 2.3. Instruments 

#### 2.3.1. Y-Balance Test

**Figure 2:**
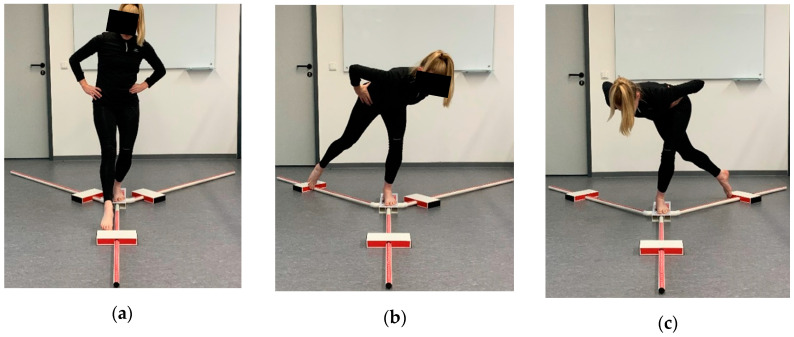
Y-Balance Test: (**a**) anterior (ANT); (**b**) posteromedial (PM); (**c**) posterolateral (PL).

Dynamic postural control was measured using the YBT (functionalmovement.com, Danville, VA, USA). The YBT trials were conducted according to the guidelines by Gribble et al. [26]. The YBT requires the subject to stand barefoot on a platform with the hands on the hips, maintaining the balance with one leg while sliding a block as far as possible in each direction (ANT, PM, PL) with the other leg (Figure 2). Thereby the dynamic postural control of the leg that is standing on the platform is examined. After sliding the block in each direction, the subjects returned to a bilateral stance. Participants then switched sides and conducted the YBT standing on the other leg. A trial was considered invalid if one of the formerly published criteria by Plisky et al. [38] was fulfilled. In such case, subjects had to start over with the current trial.

The reach distance in each direction was measured and normalized to leg length using the following equation [38]:

(1) $$\mathrm{Normalized}\;\mathrm{reach}\;\mathrm{distance}\;\left(\%\right)\;=\;\frac{\mathrm{reach}\;\mathrm{distance}\;(\mathrm{cm})}{\mathrm{leg}\;\mathrm{length}\;(\mathrm{cm})}\;\times 100$$

Moreover, a normalized composite score (CS) was computed for each leg [38]:

(2) $$\mathrm{Composite}\;\mathrm{score}\;\left(\%\right)\;=\;\frac{\mathrm{reach}\;\mathrm{distance}\;\mathrm{ANT}\;(\mathrm{cm})\;+\;\mathrm{reach}\;\mathrm{distance}\;\mathrm{PM}\;(\mathrm{cm})\;+\;\mathrm{reach}\;\mathrm{distance}\;\mathrm{PL}\;(\mathrm{cm})}{\mathrm{leg}\;\mathrm{length}\;\left(\mathrm{cm}\right)\;\times \;3}\;\times 100$$

The side-difference of the reach distances in the ANT direction was also calculated:

(3) Side-difference ANT (cm) = reach distance ANT kicking leg (cm) – reach distance ANT standing leg (cm)

#### 2.3.2. Modified Wingate Anaerobic Test

The subjects completed a modified version of the Wingate Anaerobic-Test [40] on a bicycle ergometer (Cyclus 2, RBM elektronik-automation GmbH, Leipzig, Germany). According to the study of Johnston et al. [3], the test consisted of 60 s maximal intensity cycling. Directly after the 5-min warm-up, the subjects started to cycle at a cadence of 50–60 RPM for 30 s. The Wingate protocol started directly after 30 s and subjects were instructed to accelerate and to maintain the maximal effort during the following 60 s. Resistance was set at 7.5% of the subject’s weight. Heart rate was measured during the protocol with a Polar^®^ sensor. 

### 2.4. Statistical Analyses 

The data were analyzed using SPSS (version 27.0, IBM Corporation, Armonk, New York, NY, USA) and presented as mean (M) ± standard deviation (SD). All data were checked for normal distribution through the Shapiro–Wilk test. To assess reliability, different values were used. The ICC (3, 1) with absolute agreement [45] was calculated across the three baseline measurements of the YBT to determine the repeatability of the normalized reach distances. The values were interpreted according to Koo and Li [46] as > 0.9 = excellent, 0.75–0.9 = good, 0.5–0.75 = moderate, and <0.5 = poor. Within-session reliability was assessed using the CV calculated as CV = (SD/M) × 100. CV values < 10% were acceptable according to Cormack et al. [44]. Moreover, the standard error of measurement (SEM) was calculated as SD × √1-ICC to assess the degree of variation between the repeated measures. 

Repeated measures analyses of variance (ANOVA) with a Greenhouse–Geisser correction, if necessary, were conducted to regard the differences between the pre-load and the post-load measurements (main and interaction effects) for the NRDs, CSs, and side-differences. Additional contrast analyses for repeated measures were conducted to specify the differences between adjacent points of time. Effect sizes for repeated measures (Cohen’s d_z_) and 95% CIs were calculated between the different points of time using the website psychometrica [47]. 90% CIs for η_p_^2^ were determined with the SPSS syntax by Wuensch [42]. 

## 3. Results

Regarding the injury risk of an athlete, only the values of the ANT reach direction and the normalized CSs are associated with the injury risk. Therefore, only the results concerning the ANT reach direction and the normalized CSs are reported. The results concerning the other directions are provided in a supplement, which includes tables and figures for all reach distances. 

Before the inferential statistics, it was tested that the individual reach distance (ANT) is not highly correlated with the composite score (CS) (r = 0.42 [kicking leg], r = 0.48 [standing leg], Table S1). Therefore, an examination of the single reach distance values is appropriate. The subjects achieved a heart rate from 184 ± 10 BPM immediately post-load, indicating that they were completely exhausted due to the protocol.

### 3.1. Statistical Assumptions

The ICC (3, 1) values ranged from 0.92–0.97 for the three baseline measurements of the given normalized reach distances, indicating excellent reliability. The SEM ranged between 0.35–0.76, and the CV ranged from 2.54–3.05 for the three baseline values (Table S2). Therefore, only the final pre-load measurement (pre03) was used in further analysis.

Comparing the values between the two groups with 2 × 4 repeated measures ANOVAs, no significant group differences were found for any of the variables, after alpha adjustment α* = 0.01. Therefore, the values of both groups were pooled for further analysis. 

### 3.2. Normalized Reach Distances and Composite Scores

All data were normally distributed according to West, Finch, and Curran [48], due to a given skewness < 2 and kurtosis < 7. The normalized reach distances for the ANT direction and the normalized CS are provided in Table 2. The values for the other directions are provided in Table S3.

**Figure 3:**
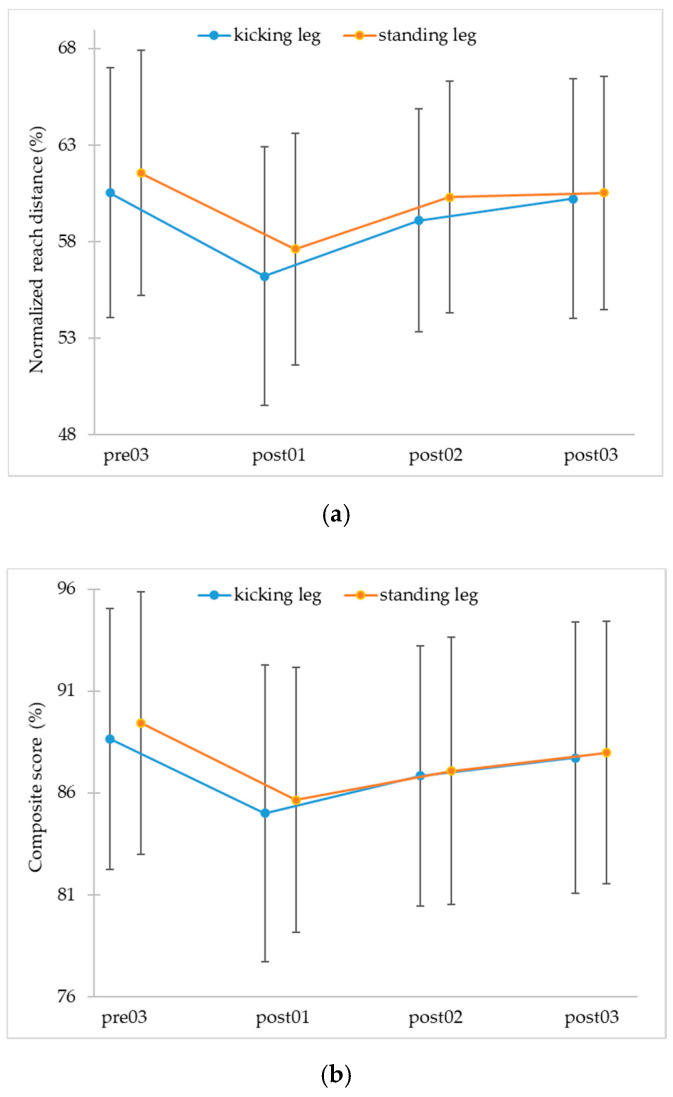
Mean values and standard deviations of (**a**) normalized reach distance anterior and (**b**) normalized composite scores at the four points of time (pre03 = pre-load, post01 = 0 min post-load, post02 = 10 min post-load, post03 = 20 min post-load).

A repeated measures ANOVA showed a statistically significant difference for the ANT direction (F_3, 189_ = 27.27, *p* < 0.001, η_p_^2^ = 0.30, 90%CI [.21, 0.37], 1-β > 0.99) and for the CS (F_3, 189_ = 17.82, *p* < 0.001, η_p_^2^= 0.22, 90% CI [0.13, 0.29], 1-β > 0.99) of the kicking leg and for the ANT direction (F_3, 189_ = 27.67, *p* < 0.001, η_p_^2^ = 0.31, 90% CI [0.21−0.38], 1-β > 0.99) and the CS (F_3, 189_ = 20.26, *p* < 0.001, η_p_^2^ = 0.24, 90% CI [0.15, 0.32], 1-β > 0.99) of the standing leg (see Figure 3; data for the other directions are provided in Figure S1 and Table S4).

A contrast analysis (Table 3) revealed expected significant changes between pre03 and post01 (contrast 1), and post01 and post02 (contrast 2) for the ANT direction and the CS in both legs. Between post02 and post03 (contrast 3), there were also statistically significant changes for the CS on both legs and the ANT direction for the kicking leg. Regarding the differences between pre03 and the final post-load (post03) measurement (contrast 4), there were statistically significant differences for the ANT direction of the standing leg and the CS in both legs. The values in ANT direction of the kicking leg were not significantly different between pre03 and post03. The results for the other directions are provided in Table S5. 

### 3.3. Side-Difference Anterior 

Side-differences in the anterior direction (cm) are shown in Table 4 and Figure S2. A repeated measures ANOVA revealed no statistically significant differences between the four points of time (F_3, 189_ = 0.21, *p* = 0.87, η_p_^2^ = 0.003, 1-β = 0.09). 

## 4. Discussion

The study aimed to investigate the influence of physical load on dynamic postural control. The results show a negative influence of physical load on dynamic postural control immediately after the loading protocol, a successive increase after the load, and a return to almost baseline levels 20 min post-load. 

The results are in line with the hypotheses. First, it was hypothesized that dynamic postural control would decrease directly after the loading protocol (H1). Such a decrease is shown for all reach directions, as well as for the composite scores. This supports the findings of Johnston et al. [3], who also found a negative effect of a Wingate Anaerobic Test on dynamic postural control measured with the YBT. The achieved heart rate values of 184 ± 10 BPM are comparable, demonstrating that the study subjects were equally stressed due to the Wingate Anaerobic Test on the bicycle ergometer. Additionally, the percentage changes of the normalized reach distances are also comparable to those of Johnston et al. [3]. Moreover, the results are also in line with other studies, showing that physical load, e.g., local fatiguing protocols [15], a HIT protocol [5], or a functional protocol [17] has detrimental effects on dynamic postural control immediately after a loading protocol measured with the YBT or SEBT. These decrements might be ascribed to changes due to the load, such as neuromuscular impairment [3,4,5,22,23,29] (e.g., reduced motor drive caused by centrally induced inhibition) and loss of sensorimotor input/control, due to changes of afferent information, as well as altered proprioception, altered joint awareness, and alterations of muscle contraction efficacy [3,18,23,24,25,26,27,28,29]. Nevertheless, compared with previous findings using similar protocols there are also differences in the results and the extent of the changes. For example, Wright et al. [20] found no effect of a cycling protocol on dynamic postural control measured with a Biodex Balance System; however, this test might challenge different aspects of dynamic balance than the YBT or SEBT. The Biodex Balance System challenges reactive postural control, meaning the “response to an external disturbance in stability” (p. 322). Tests as the SEBT or YBT are testing “actions taken in preparation for a potential destabilizing event” (p. 322), i.e., anticipatory postural control [2]. Therefore, it might be helpful to differentiate between reactive and anticipatory postural control and to regard both. Hence, dynamic postural control seems to be task-specific and this task specificity must be concerned in research. Additionally, the results seem also to depend on the type of protocol. For example, Wright et al. [20] found a negative effect after running but not after cycling, suggesting that the results may additionally depend on the type of load and the induced type of fatigue (whole body vs. local) [20]. This loading protocol specificity should also be considered during future studies [29] and the used tasks and protocols should try to mimic the sport-specific demands [33]. 

Besides the acute effect of physical load on dynamic postural control, the recovery time was also regarded, similar to the study of Johnston et al. [3]. Based on the findings of Johnston et al. [3], it was hypothesized that dynamic postural control will successively increase after the load (H2) and that it will be almost at baseline levels 20 min post-load (H3). Regarding the results, the normalized reach distances recovered within a period of 20 min in ANT direction of the kicking leg and almost recovered in ANT direction of the standing leg and CS of both legs. Nevertheless, the recovery time seems to be a little bit longer in the standing leg compared with the kicking leg. To confirm this finding and to illustrate the exact recovery times, further studies with post-testing for longer than 20 min and at more points of time are required. Moreover, the recovery time may additionally depend on the tasks used to assess dynamic postural control and the loading protocol. Wright et al. [20] and Güler et al. [21] found lower recovery times of dynamic postural control measured with the Biodex Balance System after running and cycling. These different results might also be described with the different types of tasks used, challenging different types of dynamic postural control (reactive or anticipatory). Therefore, the type of task used has to be considered in further research and both types of dynamic postural control as well as interaction with the type of load and protocol need to be regarded.

Regarding side-differences, it was expected that the changes would occur in both legs (H4) and that side-differences in ANT direction would change due to the load (H5). The results show changes of the values for both legs. However, the side-differences in anterior direction did not increase due to the load. Therefore, the changes in dynamic postural control due to physical load seem to be a global effect. Both legs were stressed equally and no side-differences occurred as it was already shown for YBT values during rest [36]. Nevertheless, the used loading protocol consisted of cycling on an ergometer, which is a largely symmetric and controlled movement. This might explain the comparable changes in both legs. The effect of more challenging loading protocols on dynamic postural control needs to be clarified because different loading protocols challenge different systems and can lead to different types of fatigue with different consequences (physiological, visual, vestibular) [20,29]. Moreover, the structure (intensity, duration, etc.) of the loading protocol might need to be changed to examine possible side-differences and to assess the influence of physical load on side-differences. Actually, the loading protocol included only one short bout of high intensity, but, for example, during matches a lot more of such bouts are present [5]. Therefore, possible changes might accumulate during these bouts and only manifest during the later stages of a match. This could explain why the number of injuries increases towards the end of a match [5,13]. Hence, loading protocols with more bouts might depict the real demands better and might reveal side-differences due to sport-specific actions.

A decrease of dynamic postural control, as found here, has previously been associated with a higher potential injury risk [3,5]. The expected neuromuscular and sensorimotor deficits might lead to changes of movements and biomechanics [3,4,5,22,25]. Therefore, the impact during dynamic actions (e.g., landing or cutting) could not be compensated [8], which then may lead to injuries such as ACL ruptures. The present results show decreased dynamic postural control directly after the load and a recovery in about 20 min. Therefore, it can be assumed that the injury risk is potentially increased until 20 min post-load. This is also supported by the findings regarding the composite scores. The number of subjects below the critical value of under 94% [9,10] is increased after the load for both legs. Only three subjects (out of 64) had no higher potential injury risk (in both legs) after the loading protocol, and 5 had an increased injury risk for only one leg. Moreover, the potential injury risk is not additionally increased due to higher side-differences. The number of subjects with critical side-differences anterior greater than 4 cm [11] did not increase after the loading protocol. However, all the subjects with a side-difference greater than 4 cm had composite scores under 94% post-load, indicating an especially high potential injury risk for these subjects. In future work, given inter-individual variation and task-specificity of side-differences, a more individual approach seems advisable [33,49]. Nevertheless, the prediction of the likelihood of injuries using the YBT has recently been doubted [50], and the particular YBT is not able to give an overall impression of all types of dynamic postural control. Therefore, researchers should also think about supplementing their assessment with other methods to examine dynamic postural control, e.g., reactive postural control. 

The current study has some limitations that must be considered. (1) The total recovery time of 20 min was not long enough to restore the NRD in ANT direction and the CS. Therefore, a longer period is recommended to estimate recovery times more precisely. (2) As in the study of Johnston et al. [3], the subjects had different sporting backgrounds and this might lead to different reactions to the loading protocol. Hence, it could be helpful to test subjects from one sport only (i) to reduce load-induced variability in dynamic postural control, (ii) to detect the sport-specific demands on dynamic postural control, and (iii) to conceptualize suitable training sessions and programs. Moreover, (3) the implemented loading protocol consisted of cycling on an ergometer, which is a symmetric and controlled movement that does not optimally reflect the sporting demands in, e.g., sport games. Loading protocols that are closer to the more asymmetric demands of sports (e.g., soccer, handball) are needed for more representative testing of dynamic postural control and possible side-differences. Considering these points in future studies may help improve understanding the effect of physical load on dynamic postural control and its association with injury risk.

Altogether, testing dynamic postural control under loading conditions can facilitate the detection of hidden neuromuscular and sensorimotor deficits, showing which athlete is at a higher potential injury risk. Therefore, testing dynamic balance under both rest and loaded conditions is recommended. Thereby the tasks and protocols used should mimic the sport-specific demands. Moreover, an additional regarding of reactive postural control might obtain further insights. Additionally, both legs should be tested to assess possible side-differences that might be apparent during loading protocols other than cycling. According to the present findings, the order in which legs are tested does not seem to play a crucial role. In addition, to obtain more insights and a comprehensive impression of an athlete, it is helpful not only to look at changes of dynamic postural control due to load but also to regard a combination of several tasks and protocols reflecting the demands of a sport and the “risky” situations.

## 5. Conclusions

The results of the current study show a negative effect of physical load on dynamic postural control, indicating a higher potential injury risk, which lasts for about 20 min post-load. Findings should be regarded during the conception of training sessions and programs, to reduce an athlete’s potential injury risk. Testing of dynamic postural control during resting and loading conditions is advisable to detect potential neuromuscular and sensorimotor deficits of an athlete. Moreover, a combination of different tests and tasks could be helpful to obtain a comprehensive impression of an athlete. The effect of loading protocols that are closer to the real demands concerning type, structure, and duration needs to be clarified and might help to detect possible side-differences and to give better insight into an athlete’s potential injury risk. 

## Figures and Tables

**Table 1:** Subject characteristics.

	Total	Group 1	Group 2
*N*	64 (16 m, 16 f)	32 (16 m, 16 f)	32 (16 m, 16 f)
Age (years) (M ± SD)	24.11 ± 2.42	24.44 ± 2.86	23.78 ± 1.88
Height (cm) (M ± SD)	175.53 ± 8.17	175.19 ± 7.9	175.87 ± 8.54
Weight (kg) (M ± SD)	67.16 ± 10.08	67.5 ± 9.32	66.88 ± 10.92
Leg length kicking leg (cm) (M ± SD)	94.94 ± 6.54	94.53 ± 5.73	95.35 ± 7.33
Leg length standing leg (cm) (M ± SD)	94.94 ± 6.59	94.53 ± 5.81	95.34 ± 7.35

**Table 2:** Mean normalized reach distances (ANT) and normalized composite scores (CS).

		pre03	post01	post02	post03
ANT kicking leg	M ± SD	60.53 ± 6.48	56.22 ± 6.70	59.10 ± 5.77	60.23 ± 6.22
	d_z_^1^ [95% CI]		−0.82 [−1.20, −0.47]	0.68 [0.28, 0.99]	0.32 [−0.02, 0.68]
	Average change ^2^ (M ± SD)		4.32 ± 5.25	1.43 ± 3.75	0.31 ± 3.57
	Change (%) ^2^		−7.14	−2.37	−0.51
ANT standing leg	M ± SD	61.56 ± 6.34	57.62 ± 5.99	60.31 ± 6.00	60.53 ± 6.05
	d_z_^1^ [95% CI]		−0.83 [−1.17, −0.45]	0.79 [0.43, 1.15]	0.09 [−0.26, 0.44]
	Average change ^2^ (M ± SD)		3.94 ± 4.77	1.25 ± 3.18	1.03 ± 3.38
	Change (%) ^2^		−6.40	−2.03	−1.67
CS kicking leg	M ± SD	88.66 ± 6.40	85.01 ± 7.28	86.84 ± 6.38	87.73 ± 6.64
	d_z_^1^ [95% CI]		−0.67 [−1.08, −0.36]	0.46 [0.08, 0.78]	0.30 [−0.04, 0.66]
	Average change ^2^ (M ± SD)		3.66 ± 5.49	1.82 ± 3.98	0.93 ± 3.56
	Change (%) ^2^		−4.13	−2.05	−1.05
CS standing leg	M ± SD	89.43 ± 6.43	85.66 ± 6.48	87.08 ± 6.55	87.97 ± 6.44
	d_z_^1^ [95% CI]		−0.71 [−1.07, −0.35]	0.44 [0.09, 0.79]	0.32 [−0.04, 0.66]
	Average change ^2^ (M ± SD)		3.76 ± 5.33	2.35 ± 4.18	1.46 ± 3.68
	Change (%) ^2^		−4.21	−2.62	−1.63

ANT = anterior; CI = confidence interval; CS = composite score; pre03 = pre-load; post01 = 0 min post-load; post02 = 10 min post load; post03 = 20 min post load. ^1^ Compared to the previous point of time; ^2^ compared to pre03.

**Table 3:** Results of the contrast analysis.

			Between		F (1, 63)	*p*	η_p_^2^	1-β
Normalized values (%)	ANT kicking leg	Contrast 1	Pre03	Post01	43.34	<0.001	0.41	>0.99
		Contrast 2	Post 01	Post02	28.87	<0.001	0.31	>0.99
		Contrast 3	Post02	Post03	6.39	0.01	0.09	0.70
		Contrast 4	Pre03	Post03	0.05	0.49	0.01	0.11
	ANT standing leg	Contrast 1	Pre03	Post01	43.68	<0.001	0.41	>0.99
		Contrast 2	Post 01	Post02	39.68	<0.001	0.09	>0.99
		Contrast 3	Post02	Post03	0.52	0.47	0.01	0.11
		Contrast 4	Pre03	Post03	5.92	0.02	0.09	0.67
	CS kicking leg	Contrast 1	Pre03	Post01	28.44	<0.001	0.31	>0.99
		Contrast 2	Post 01	Post02	13.17	0.001	0.17	0.95
		Contrast 3	Post02	Post03	5.82	0.02	0.09	0.66
		Contrast 4	Pre03	Post03	4.42	0.04	0.07	0.54
	CS standing leg	Contrast 1	Pre03	Post01	31.92	<0.001	0.34	>0.99
		Contrast 2	Post 01	Post02	12.23	0.001	0.16	0.93
		Contrast 3	Post02	Post03	6.39	0.01	0.09	0.70
		Contrast 4	Pre03	Post03	10.03	0.002	0.14	0.88

ANT = anterior; CS = composite score; pre03 = pre-load; post01 = 0 min post-load; post02 = 10 min post-load; post03 = 20 min post-load.

**Table 4:** Side-differences anterior (cm).

	pre03	post01	post02	post03
M ± SD	3.52 ± 3.16	3.52 ± 2.67	3.33 ± 2.89	3.28 ± 2.77
d_z_^1^ [95% CI]		0 [−0.35, 0.35]	−0.07 [−0.42, 0.28]	−0.02 [−0.36, 0.33]
Average change (cm) ^2^ (M ± SD)		0 ± 3.42	0.19 ± 2.42	0.23 ± 2.95
Change (%) ^2^		0	−5.33	−6.67

pre03 = pre-load; post01 = 0 min post-load; post02 = 10 min post-load; post03 = 20 min post-load. ^1^ Compared to the previous point of time; ^2^ compared to pre03.

## Supplementary Materials

The following are available online at https://www.mdpi.com/2411-5142/5/4/100/s1, Figure S1: Mean normalized values and standard deviations for (**a**) anterior, (**b**) posteromedial, (**c**) posterolateral, and (**d**) composite scores at the four points of time; Figure S2: Mean side-differences anterior and standard deviation at the four points of time; Table S1: Correlation with the composite score; Table S2: Reliability analysis (Pre01, Pre02, Pre03); Table S3: Mean normalized reach distances and normalized composite scores; Table S4: Results of the repeated measures ANOVA. Table S5: Results of the contrast analysis. A spreadsheet of the full data will also be available in the supplementary material.
