# Supplementary material for: The Influence of Physical Load on Dynamic Postural Control—A Systematic Replication Study
Source: J Funct Morphol Kinesiol. 2020 Dec 21;5(4):100. doi: 10.3390/jfmk5040100 (PMC7804868; doi:10.3390/jfmk5040100)
Supplement: Supplementary file 1 [file jfmk-05-00100-s001.zip › Supplementary figures.pdf]

Supplementary Figures

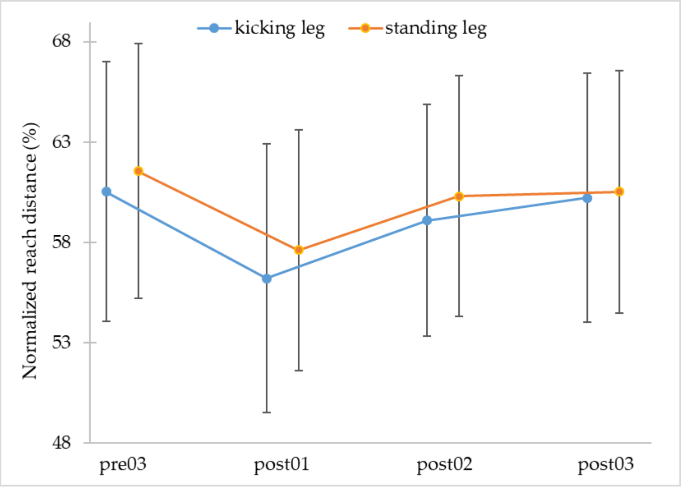

(**a**)

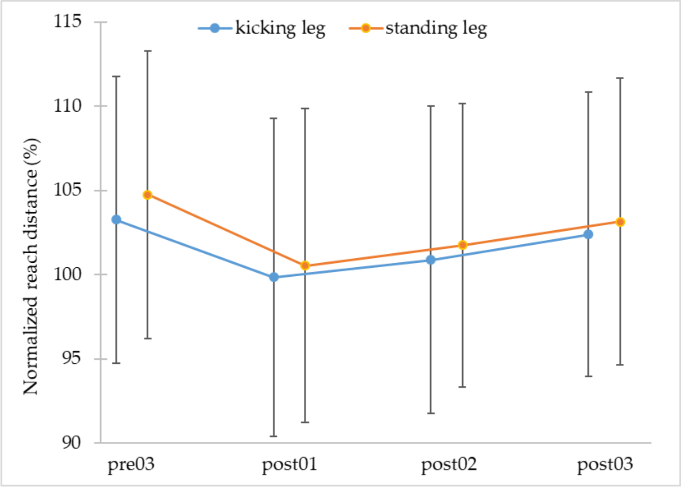

(**b**)

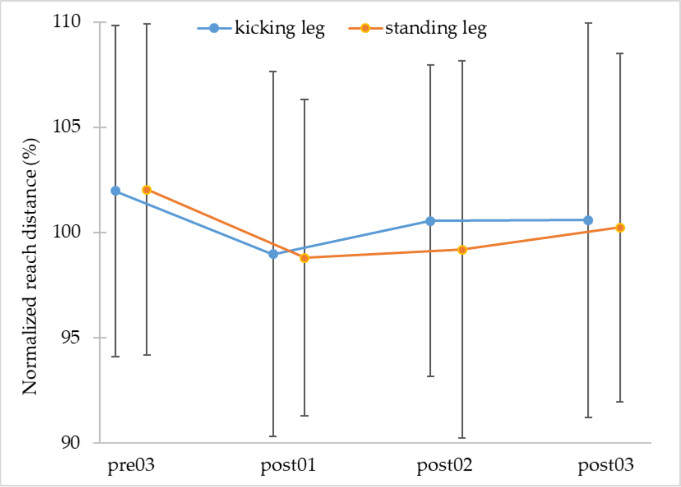

(**c**)

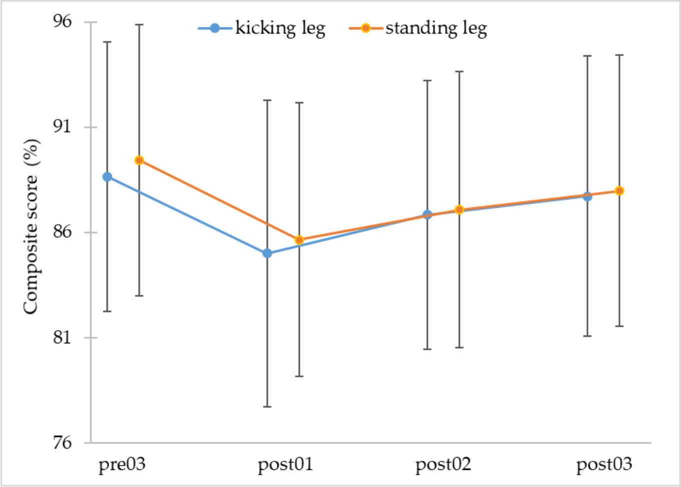

(**d**)

Figure S1. Mean normalized values and standard deviations (**a**) anterior, (**b**) posteromedial, (**c**) posterolateral and (**d**) composite scores at the four points of time (pre03 = pre-load; post01 = 0 min post-load; post02 = 10 min post-load; post03 = 20 min post-load).

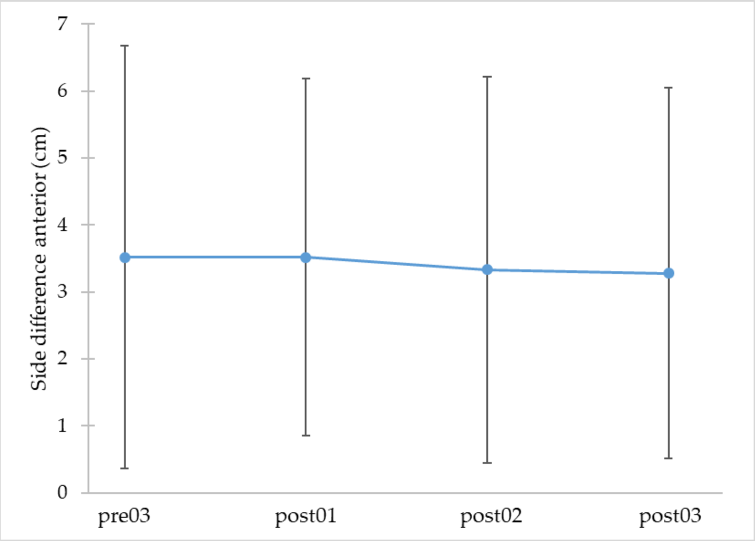

Figure S2. Mean side-differences anterior and standard deviation at the four points of time (pre03 = pre-load; post01 = 0 min post-load; post02 = 10 min post-load; post03 = 20 min post-load).
