# Supplementary material for: The Influence of Physical Load on Dynamic Postural Control—A Systematic Replication Study
Source: J Funct Morphol Kinesiol. 2020 Dec 21;5(4):100. doi: 10.3390/jfmk5040100 (PMC7804868; doi:10.3390/jfmk5040100)
Supplement: Supplementary file 1 [file jfmk-05-00100-s001.zip › Supplementary tables.docx]

**Supplementary Tables**

**Table S1.** Correlation with the composite score.

|  | r |
| --- | --- |
| ANT kicking leg | .42 |
| ANT standing leg | .48 |
| PM kicking leg | .57 |
| PM standing leg | .56 |
| PL kicking leg | .48 |
| PL standing leg | .40 |
| ANT = anterior; PM = posteromedial; PL = posterolateral; | |

**Table S2.** Reliability analysis (Pre01, Pre02, Pre03).

|  | **ICC** (3,1) | **SEM (**SD × √1-ICC) | **CV** (SD/mean) × 100 |
| --- | --- | --- | --- |
| ANT  kicking leg | .96  95% CI [.94, .98] | 0.35 | 3.05 |
| ANT  standing leg | .97  95% CI [.95, .98] | 0.30 | 2.65 |
| PM  kicking leg | .95  95% CI [.93, .97] | 0.55 | 2.54 |
| PM  standing leg | .93  95% CI [.89, .96] | 0.77 | 2.86 |
| PL  kicking leg | .93  95% CI [.89, .96] | 0.71 | 2.70 |
| PL  standing leg | .92  95% CI [.88, .95] | 0.75 | 2.66 |
| ANT = anterior; CI = confidence interval; CV = coefficient of variation; ICC = intraclass correlation coefficient; PM = posteromedial; PL = posterolateral; SD = standard deviation; SEM = standard error of measurement | | | |

**Table S3.** Mean normalized reach distances and normalized composite scores.

|  |  | **pre03** | **post01** | **post02** | **post03** |
| --- | --- | --- | --- | --- | --- |
| ANT  kicking leg | M ± SD | 60.53 ± 6.48 | 56.22 ± 6.70 | 59.10 ± 5.77 | 60.23 ± 6.22 |
|  | d_z_^1^  [95% CI] |  | -0.82  [-1.20, -0.47] | 0.68  [0.28, 0.99] | 0.32  [-0.02, 0.68] |
|  | Average change^2^  (M ± SD) |  | 4.32 ± 5.25 | 1.43 ± 3.75 | 0.31 ± 3.57 |
|  | Change (%)^2^ |  | -7.14 | -2.37 | -0.51 |
| ANT  standing leg | M ± SD | 61.56 ± 6.34 | 57.62 ± 5.99 | 60.31 ± 6.00 | 60.53 ± 6.05 |
|  | d_z_^1^  [95% CI] |  | -0.83  [-1.17, -0.45] | 0.79  [0.43, 1.15] | 0.09  [-0.26, 0.44] |
|  | Average change^2^  (M ± SD) |  | 3.94 ± 4.77 | 1.25 ± 3.18 | 1.03 ± 3.38 |
|  | Change (%)^2^ |  | -6.40 | -2.03 | -1.67 |
| PM  kicking leg | M ± SD | 103.27 ± 8.51 | 99.84 ± 9.45 | 100.88 ± 9.12 | 102.38 ± 8.43 |
|  | d_z_^1^  [95% CI] |  | -0.50  [-0.88, -0.17] | 0.17  [-0.19, 0.51] | 0.39  [0.02, 0.72] |
|  | Average change^2^  (M ± SD) |  | 3.43 ± 6.97 | 2.39 ± 6.15 | 0.89 ± 4.58 |
|  | Change (%)^2^ |  | -3.32 | -2.32 | -0.86 |
| PM  standing leg | M ± SD | 104.74 ± 8.52 | 100.53 ± 9.32 | 101.75 ± 8.41 | 103.15 ± 8.50 |
|  | d_z_^1^  [95% CI] |  | -0.61  [-1.0, -0.29] | 0.23  [-0.12, 0.57] | 0.30  [-0.04, 0.65] |
|  | Average change^2^  (M ± SD) |  | 4.21 ± 6.91 | 2.98 ± 5.85 | 1.58 ± 5.20 |
|  | Change (%)^2^ |  | -4.02 | -2.85 | -1.51 |
| PL  kicking leg | M ± SD | 101.97 ± 7.85 | 98.97 ± 8.66 | 100.55 ± 7.40 | 100.58 ± 9.37 |
|  | d_z_^1^  [95% CI] |  | -0.38  [-0.75, -0.05] | 0.26  [-0.10, 0.59] | 0.01  [-0.34, 0.35] |
|  | Average change^2^  (M ± SD) |  | 3.01 ± 7.84 | 1.43 ± 6.04 | 1.39 ± 6.44 |
|  | Change (%)^2^ |  | -2.95 | -1.40 | -1.37 |
| PL  standing leg | M ± SD | 102.04 ± 7.87 | 98.80 ± 7.51 | 99.19 ± 8.95 | 100.23 ± 8.26 |
|  | d_z_^1^  [95% CI] |  | -0.44  [-0.78, -0.08] | 0.07  [-0.27, 0.43] | 0.21  [-0.14, 0.55] |
|  | Average change^2^  (M ± SD) |  | 3.24 ± 7.33 | 2.85 ± 6.21 | 1.81 ± 5.47 |
|  | Change (%)^2^ |  | -3.18 | -2.80 | -1.77 |

**Table S3.** Continued.

|  | | **pre03** | **post01** | **post02** | **post03** |
| --- | --- | --- | --- | --- | --- |
| CS  kicking leg | M ± SD | 88.66 ± 6.40 | 85.01 ± 7.28 | 86.84 ± 6.38 | 87.73 ± 6.64 |
|  | d_z_^1^  [95% CI] |  | -0.67  [-1.08, -0.36] | 0.46  [0.08, 0.78] | 0.30  [-0.04, 0.66] |
|  | Average change^2^  (M ± SD) |  | 3.66 ± 5.49 | 1.82 ± 3.98 | 0.93 ± 3.56 |
|  | Change (%)^2^ |  | -4.13 | -2.05 | -1.05 |
| CS  standing leg | M ± SD | 89.43 ± 6.43 | 85.66 ± 6.48 | 87.08 ± 6.55 | 87.97 ± 6.44 |
|  | d_z_^1^  [95% CI] |  | -0.71  [-1.07, -0.35] | 0.44  [0.09, 0.79] | 0.32  [-0.04, 0.66] |
|  | Average change^2^  (M ± SD) |  | 3.76 ± 5.33 | 2.35 ± 4.18 | 1.46 ± 3.68 |
|  | Change (%)^2^ |  | -4.21 | -2.62 | -1.63 |
| ANT = anterior; CI = confidence interval; CS = composite score; PM = posteromedial; PL = posterolateral; pre03 = pre-load; post01 = 0 min post-load; post02 = 10 min post load; post03 = 20 min post load | | | | | |

^1^compared to the previous point of time; ^2^compared to pre03

**Table S4.** Results of the repeated measures ANOVA.

|  |  | **F (3, 189)** | **p** | **η_p_^2^** | **1-β** |
| --- | --- | --- | --- | --- | --- |
| Normalized values (%) | ANT  kicking leg | 27.27 | < .001 | .30 | > .99 |
|  | ANT  standing leg | 27.67 | < .001 | .31 | > .99 |
|  | PM  kicking leg | 9.11 | < .001 | .13 | .99 |
|  | PM  standing leg | 13.36 | < .001 | .18 | > .99 |
|  | PL  kicking leg | 4.25 | .01 | .06 | .81 |
|  | PL  standing leg | 7.61 | < .001 | .11 | .97 |
|  | CS  kicking leg | 17.82 | < .001 | .22 | > .99 |
|  | CS  standing leg | 20.26 | < .001 | .24 | > .99 |
| Side-difference anterior (cm) | | 0.21 | .87 | < .01 | .09 |
| ANT = anterior; CS = composite score; PM = posteromedial, PL = posterolateral | | | | | |

**Table S5.** Results of the contrast analysis.

|  | | |  | **Between** | | **F (1, 63)** | **p** | **η_p_^2^** | **1-β** |
| --- | --- | --- | --- | --- | --- | --- | --- | --- | --- |
| Normalized values (%) | ANT  kicking leg | | Contrast 1 | Pre03 | Post01 | 43.34 | < .001 | .41 | > .99 |
|  |  |  | Contrast 2 | Post 01 | Post02 | 28.87 | < .001 | .31 | > .99 |
|  |  |  | Contrast 3 | Post02 | Post03 | 6.39 | .01 | .09 | .70 |
|  |  |  | Contrast 4 | Pre03 | Post03 | 0.05 | .49 | .01 | .11 |
|  | ANT  standing leg | | Contrast 1 | Pre03 | Post01 | 43.68 | < .001 | .41 | > .99 |
|  |  |  | Contrast 2 | Post 01 | Post02 | 39.68 | < .001 | .09 | > .99 |
|  |  |  | Contrast 3 | Post02 | Post03 | 0.52 | .47 | .01 | .11 |
|  |  |  | Contrast 4 | Pre03 | Post03 | 5.92 | .02 | .09 | .67 |
|  | PM  kicking leg | | Contrast 1 | Pre03 | Post01 | 15.48 | < .001 | .20 | .97 |
|  |  |  | Contrast 2 | Post 01 | Post02 | 1.73 | .19 | .03 | .25 |
|  |  |  | Contrast 3 | Post02 | Post03 | 9.38 | .003 | .13 | .85 |
|  |  |  | Contrast 4 | Pre03 | Post03 | 2.41 | .13 | .04 | .33 |
|  | PM  standing leg | | Contrast 1 | Pre03 | Post01 | 23.69 | < .001 | .27 | > .99 |
|  |  |  | Contrast 2 | Post 01 | Post02 | 3.44 | .07 | .05 | .45 |
|  |  |  | Contrast 3 | Post02 | Post03 | 5.94 | .02 | .09 | .67 |
|  |  |  | Contrast 4 | Pre03 | Post03 | 5.95 | .02 | .09 | .67 |
|  | PL  kicking leg | | Contrast 1 | Pre03 | Post01 | 9.43 | .003 | .13 | .86 |
|  |  |  | Contrast 2 | Post 01 | Post02 | 4.27 | .04 | .06 | .53 |
|  |  |  | Contrast 3 | Post02 | Post03 | < 0.01 | .97 | < .01 | .05 |
|  |  |  | Contrast 4 | Pre03 | Post03 | 3.00 | .09 | .05 | .40 |
|  | PL  standing leg | | Contrast 1 | Pre03 | Post01 | 12.53 | .001 | .17 | .94 |
|  |  |  | Contrast 2 | Post 01 | Post02 | 0.32 | .57 | .01 | .09 |
|  |  |  | Contrast 3 | Post02 | Post03 | 2.93 | .04 | .04 | .39 |
|  |  |  | Contrast 4 | Pre03 | Post03 | 6.98 | .01 | .10 | .74 |
|  | CS  kicking leg | | Contrast 1 | Pre03 | Post01 | 28.44 | < .001 | .31 | > .99 |
|  |  |  | Contrast 2 | Post 01 | Post02 | 13.17 | .001 | .17 | .95 |
|  |  |  | Contrast 3 | Post02 | Post03 | 5.82 | .02 | .09 | .66 |
|  |  |  | Contrast 4 | Pre03 | Post03 | 4.42 | .04 | .07 | .54 |
|  | CS standing leg | | Contrast 1 | Pre03 | Post01 | 31.92 | < .001 | .34 | > .99 |
|  |  |  | Contrast 2 | Post 01 | Post02 | 12.23 | .001 | .16 | .93 |
|  |  |  | Contrast 3 | Post02 | Post03 | 6.39 | .01 | .09 | .70 |
|  |  |  | Contrast 4 | Pre03 | Post03 | 10.03 | .002 | .14 | .88 |
| Side-difference anterior (cm) | | | Contrast 1 | Pre03 | Post01 | < 0.01 | > .99 | < .01 | .05 |
|  |  |  | Contrast 2 | Post 01 | Post02 | 0.30 | .59 | .01 | .08 |
|  |  |  | Contrast 3 | Post02 | Post03 | 0.02 | .90 | < .01 | .05 |
|  |  |  | Contrast 4 | Pre03 | Post03 | 0.40 | .53 | .01 | .10 |
|  | | ANT = anterior; CS = composite score; pre03 = pre-load; post01 = 0 min post-load; post02 = 10 min post load; post03 = 20 min post load | | | | | | | |
