# Supplementary material for: The Influence of Physical Load on Dynamic Postural Control—A Systematic Replication Study
Source: J Funct Morphol Kinesiol. 2020 Dec 21;5(4):100. doi: 10.3390/jfmk5040100 (PMC7804868; doi:10.3390/jfmk5040100)
Supplement: Supplementary file 1 [file jfmk-05-00100-s001.zip › Supplementary tables.pdf]

Supplementary Tables

Table S1. Correlation with the composite score.

	r
ANT kicking leg	.42
ANT standing leg	.48
PM kicking leg	.57
PM standing leg	.56
PL kicking leg	.48
PL standing leg	.40

ANT = anterior; PM = posteromedial; PL = posterolateral;

Table S2. Reliability analysis (Pre01, Pre02, Pre03).

	**ICC** (3,1)	**SEM (**SD × √1-ICC)	**CV** (SD/mean) × 100
ANT kicking leg	.96 95% CI [.94, .98]	0.35	3.05
ANT standing leg	.97 95% CI [.95, .98]	0.30	2.65
PM kicking leg	.95 95% CI [.93, .97]	0.55	2.54
PM standing leg	.93 95% CI [.89, .96]	0.77	2.86
PL kicking leg	.93 95% CI [.89, .96]	0.71	2.70
PL standing leg	.92 95% CI [.88, .95]	0.75	2.66

ANT = anterior; CI = confidence interval; CV = coefficient of variation; ICC = intraclass correlation coefficient; PM = posteromedial; PL = posterolateral; SD = standard deviation; SEM = standard error of measurement

Table S3. Mean normalized reach distances and normalized composite scores.

		pre03	post01	post02	post03
ANT kicking leg	M ± SD	60.53 ± 6.48	56.22 ± 6.70	59.10 ± 5.77	60.23 ± 6.22
	d_z[1] [95% CI]		-0.82 [-1.20, -0.47]	0.68 [0.28, 0.99]	0.32 [-0.02, 0.68]
	Average change[2] (M ± SD)		4.32 ± 5.25	1.43 ± 3.75	0.31 ± 3.57
	Change (%)[2]		-7.14	-2.37	-0.51
ANT standing leg	M ± SD	61.56 ± 6.34	57.62 ± 5.99	60.31 ± 6.00	60.53 ± 6.05
	d_z[1] [95% CI]		-0.83 [-1.17, -0.45]	0.79 [0.43, 1.15]	0.09 [-0.26, 0.44]
	Average change[2] (M ± SD)		3.94 ± 4.77	1.25 ± 3.18	1.03 ± 3.38
	Change (%)[2]		-6.40	-2.03	-1.67
PM kicking leg	M ± SD	103.27 ± 8.51	99.84 ± 9.45	100.88 ± 9.12	102.38 ± 8.43
	d_z[1] [95% CI]		-0.50 [-0.88, -0.17]	0.17 [-0.19, 0.51]	0.39 [0.02, 0.72]
	Average change[2] (M ± SD)		3.43 ± 6.97	2.39 ± 6.15	0.89 ± 4.58
	Change (%)[2]		-3.32	-2.32	-0.86
PM standing leg	M ± SD	104.74 ± 8.52	100.53 ± 9.32	101.75 ± 8.41	103.15 ± 8.50
	d_z[1] [95% CI]		-0.61 [-1.0, -0.29]	0.23 [-0.12, 0.57]	0.30 [-0.04, 0.65]
	Average change[2] (M ± SD)		4.21 ± 6.91	2.98 ± 5.85	1.58 ± 5.20
	Change (%)[2]		-4.02	-2.85	-1.51
PL kicking leg	M ± SD	101.97 ± 7.85	98.97 ± 8.66	100.55 ± 7.40	100.58 ± 9.37
	d_z[1] [95% CI]		-0.38 [-0.75, -0.05]	0.26 [-0.10, 0.59]	0.01 [-0.34, 0.35]
	Average change[2] (M ± SD)		3.01 ± 7.84	1.43 ± 6.04	1.39 ± 6.44
	Change (%)[2]		-2.95	-1.40	-1.37
PL standing leg	M ± SD	102.04 ± 7.87	98.80 ± 7.51	99.19 ± 8.95	100.23 ± 8.26
	d_z[1] [95% CI]		-0.44 [-0.78, -0.08]	0.07 [-0.27, 0.43]	0.21 [-0.14, 0.55]
	Average change[2] (M ± SD)		3.24 ± 7.33	2.85 ± 6.21	1.81 ± 5.47
	Change (%)[2]		-3.18	-2.80	-1.77

Table S3. Continued.

		pre03	post01	post02	post03
CS kicking leg	M ± SD	88.66 ± 6.40	85.01 ± 7.28	86.84 ± 6.38	87.73 ± 6.64
	d_z[1]		-0.67	0.46	0.30
	[95% CI]		[-1.08, -0.36]	[0.08, 0.78]	[-0.04, 0.66]
	Average change[2] (M ± SD)		3.66 ± 5.49	1.82 ± 3.98	0.93 ± 3.56
	Change (%)[2]		-4.13	-2.05	-1.05
CS standing leg	M ± SD	89.43 ± 6.43	85.66 ± 6.48	87.08 ± 6.55	87.97 ± 6.44
	d_z[1]		-0.71	0.44	0.32
	[95% CI]		[-1.07, -0.35]	[0.09, 0.79]	[-0.04, 0.66]
	Average change[2] (M ± SD)		3.76 ± 5.33	2.35 ± 4.18	1.46 ± 3.68
	Change (%)[2]		-4.21	-2.62	-1.63

ANT = anterior; CI = confidence interval; CS = composite score; PM = posteromedial; PL = posterolateral; pre03 = pre-load; post01 = 0 min post-load; post02 = 10 min post load; post03 = 20 min post load

[1]compared to the previous point of time; [2]compared to pre03

Table S4. Results of the repeated measures ANOVA.

		**F (3, 189)**	**p**	**η_p^2**	**1-β**
Normalized values (%)	ANT kicking leg	27.27	< .001	.30	> .99
	ANT standing leg	27.67	< .001	.31	> .99
	PM kicking leg	9.11	< .001	.13	.99
	PM standing leg	13.36	< .001	.18	> .99
	PL kicking leg	4.25	.01	.06	.81
	PL standing leg	7.61	< .001	.11	.97
	CS kicking leg	17.82	< .001	.22	> .99
	CS standing leg	20.26	< .001	.24	> .99
Side-difference anterior (cm)		0.21	.87	< .01	.09

ANT = anterior; CS = composite score; PM = posteromedial, PL = posterolateral

Table S5. Results of the contrast analysis.

			Between		F (1, 63)	p	η_p^2	$1-\beta$
Normalized values (%)	ANT kicking leg	Contrast 1	Pre03	Post01	43.34	< .001	.41	> .99
		Contrast 2	Post 01	Post02	28.87	< .001	.31	> .99
		Contrast 3	Post02	Post03	6.39	.01	.09	.70
		Contrast 4	Pre03	Post03	0.05	.49	.01	.11
	ANT standing leg	Contrast 1	Pre03	Post01	43.68	< .001	.41	> .99
		Contrast 2	Post 01	Post02	39.68	< .001	.09	> .99
		Contrast 3	Post02	Post03	0.52	.47	.01	.11
		Contrast 4	Pre03	Post03	5.92	.02	.09	.67
	PM kicking leg	Contrast 1	Pre03	Post01	15.48	< .001	.20	.97
		Contrast 2	Post 01	Post02	1.73	.19	.03	.25
		Contrast 3	Post02	Post03	9.38	.003	.13	.85
		Contrast 4	Pre03	Post03	2.41	.13	.04	.33
	PM standing leg	Contrast 1	Pre03	Post01	23.69	< .001	.27	> .99
		Contrast 2	Post 01	Post02	3.44	.07	.05	.45
		Contrast 3	Post02	Post03	5.94	.02	.09	.67
		Contrast 4	Pre03	Post03	5.95	.02	.09	.67
	PL kicking leg	Contrast 1	Pre03	Post01	9.43	.003	.13	.86
		Contrast 2	Post 01	Post02	4.27	.04	.06	.53
		Contrast 3	Post02	Post03	< 0.01	.97	< .01	.05
		Contrast 4	Pre03	Post03	3.00	.09	.05	.40
	PL standing leg	Contrast 1	Pre03	Post01	12.53	.001	.17	.94
		Contrast 2	Post 01	Post02	0.32	.57	.01	.09
		Contrast 3	Post02	Post03	2.93	.04	.04	.39
		Contrast 4	Pre03	Post03	6.98	.01	.10	.74
	CS kicking leg	Contrast 1	Pre03	Post01	28.44	< .001	.31	> .99
		Contrast 2	Post 01	Post02	13.17	.001	.17	.95
		Contrast 3	Post02	Post03	5.82	.02	.09	.66
		Contrast 4	Pre03	Post03	4.42	.04	.07	.54
	CS standing leg	Contrast 1	Pre03	Post01	31.92	< .001	.34	> .99
		Contrast 2	Post 01	Post02	12.23	.001	.16	.93
		Contrast 3	Post02	Post03	6.39	.01	.09	.70
		Contrast 4	Pre03	Post03	10.03	.002	.14	.88
Side-difference anterior (cm)		Contrast 1	Pre03	Post01	< 0.01	> .99	< .01	.05
		Contrast 2	Post 01	Post02	0.30	.59	.01	.08
		Contrast 3	Post02	Post03	0.02	.90	< .01	.05
		Contrast 4	Pre03	Post03	0.40	.53	.01	.10

ANT = anterior; CS = composite score; pre03 = pre-load; post01 = 0 min post-load; post02 = 10 min post load; post03 = 20 min post load
